# From Technical Pitfall to Clinical Consequences: Leadless Pacing as a Rescue Solution

**DOI:** 10.3390/reports8040206

**Published:** 2025-10-17

**Authors:** Fulvio Cacciapuoti, Ciro Mauro, Flavia Casolaro, Antonio Torsi, Salvatore Crispo, Mario Volpicelli

**Affiliations:** 1 Division of Cardiology, “A. Cardarelli” Hospital, 80131 Naples, Italysalvatore.crispo@aocardarelli.it (S.C.); 2 Department of Advanced Medical and Surgical Sciences, “L. Vanvitelli” University, 80138 Naples, Italy; 3 Division of Arrhythmology, “Santa Maria della Pietà” Hospital, 80035 Naples, Italy; m.volpicelli@aslnapoli3sud.it

**Keywords:** pacemaker lead failure, atrial fibrillation, stroke, leadless pacemaker, venous occlusion, anticoagulation

## Abstract

**Background and Clinical Significance:** Early lead failure after dual-chamber pacemaker implantation is rare but clinically significant, particularly when associated with thromboembolic complications. Technical pitfalls at the time of implantation, such as suture fixation without protective sleeves, may be predisposed to premature lead damage and abrupt device malfunction. This case highlights the role of device interrogation in diagnosing arrhythmia-related stroke, the challenges of reimplantation in the setting of venous occlusion and anticoagulation, and the value of leadless pacing as a safe rescue strategy. **Case Presentation:** A 78-year-old man with a history of complete atrioventricular block underwent dual-chamber pacemaker implantation one year earlier. He presented to the emergency department with acute aphasia, right-sided hemiparesis, and facial asymmetry. Stroke was diagnosed, and new-onset atrial fibrillation was documented. Device interrogation revealed an abrupt fall in lead impedance followed by a sharp rise consistent with lead insulation failure and premature battery depletion. Fluoroscopy demonstrated multiple focal narrowings of the leads and complete left subclavian vein occlusion, making conventional transvenous reimplantation unfeasible, while extraction was judged high risk. Right-sided reimplantation was avoided due to hemorrhagic risk under anticoagulation. A leadless pacemaker was implanted successfully in the apico-septal region of the right ventricle via ultrasound-guided femoral access. Hemostasis was secured with a figure-of-8 suture fixed inside a 3-way tap, providing constant compression and preventing hematoma. At two-months follow-up, device function was stable and neurological recovery was favorable (mRS = 2). **Conclusions:** This case underscores how multiple adverse factors—stroke, arrhythmia detection, early device failure, venous occlusion, and anticoagulation—may converge in a single patient, and demonstrates leadless pacing as a safe and effective rescue strategy in such complex scenarios.

## 1. Introduction and Clinical Significance

Dual-chamber pacemakers are an established therapy for the management of bradyarrhythmias, particularly in patients with atrioventricular conduction disturbances [1]. By preserving atrioventricular synchrony, these devices improve cardiac output, exercise tolerance, and quality of life [2]. Modern systems are highly reliable, yet complications can still occur, often as a result of technical issues at the time of implantation [3]. Small details, such as the proper placement of sutures or the use of protective sleeves, may appear minor during the procedure but can have long-term consequences on lead integrity. Mechanical stress, insulation damage, and conductor fracture are well-documented outcomes of such technical pitfalls, and while they may remain silent for months, they can ultimately manifest as sudden lead failure or premature device depletion [4].

Another dimension in the management of patients with pacemakers is the detection of arrhythmias, including atrial fibrillation (AF). Device interrogation has become a powerful diagnostic tool, capable not only of identifying technical dysfunctions but also of uncovering arrhythmic events that may otherwise remain asymptomatic [5]. The recognition of AF is particularly relevant because of its established role as a major risk factor for ischemic stroke. Early detection of AF burden provides clinicians with an opportunity to initiate anticoagulation in accordance with international guidelines, thereby significantly reducing the risk of thromboembolic events [6].

In situations where lead revision becomes necessary, venous access problems or anatomical limitations may preclude conventional transvenous reimplantation [7]. Leadless pacemakers have emerged as a valuable alternative in this setting. Implanted directly into the right ventricle through femoral venous access, these devices bypass the need for leads and subcutaneous pockets, reducing the risks of infection, lead fracture, and hematoma [8]. Furthermore, registry data support the safety of leadless implantation in patients on uninterrupted oral anticoagulation, a feature of particular relevance in those with recent thromboembolic stroke [9].

Clinical Significance: The following case illustrates not only how technical pitfalls can result in unusually early device failure but also how a rare convergence of complications—stroke related to new atrial fibrillation, complete venous occlusion, and the need for uninterrupted anticoagulation—created an exceptionally complex therapeutic scenario. In this setting, leadless pacing provided the only safe and effective solution.

## 2. Case Presentation

A 78-year-old man with a history of hypertension, type 2 diabetes mellitus, and coronary artery disease underwent dual-chamber pacemaker implantation one year earlier for complete atrioventricular block. He presented to the emergency department with sudden onset of aphasia, right hemiparesis, and mouth angle deviation. Neurological examination confirmed a disabling focal deficit, with a National Institutes of Health Stroke Scale (NIHSS) score of 12. Urgent brain computed tomography (CT) excluded intracranial hemorrhage, and CT angiography revealed an occlusion of a left middle cerebral artery branch. The patient presented outside the therapeutic window for intravenous thrombolysis or mechanical thrombectomy. He was managed conservatively with acute stroke care and subsequently initiated on secondary prevention, including oral anticoagulation (Apixaban 5 mg twice daily) and optimization of cardiovascular risk factors.

**Figure 1:**
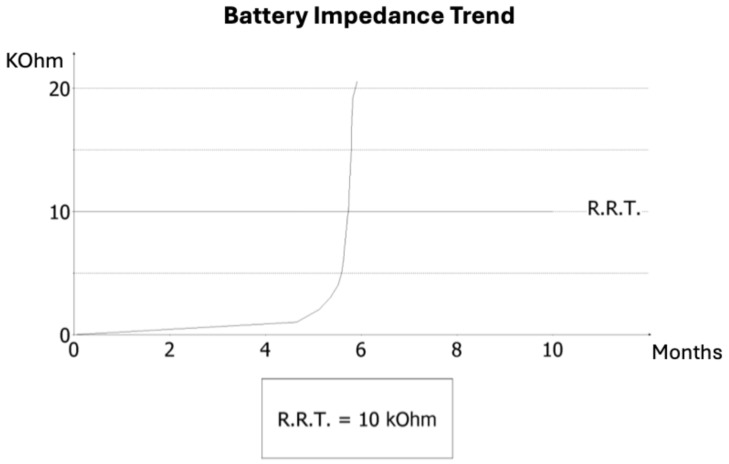
Battery impedance trend of the dual-chamber pacemaker. A rapid increase in impedance occurred around the sixth month, crossing the Recommended Replacement Time (RRT) threshold of 10 kΩ. The abrupt slope is consistent with lead-related dysfunction and explains the premature battery depletion observed in this patient.

On admission, AF was documented for the first time. Pacemaker interrogation was performed to clarify arrhythmic burden and device status. Analysis revealed that AF had been persistent for several weeks prior to admission, temporally consistent with the occurrence of the cerebrovascular event. Additionally, diagnostic review identified striking electrical abnormalities: three months earlier, the ventricular lead impedance had dropped abruptly to 145 Ω, suggesting insulation breach or conductor damage, followed by a sharp rise to values above 30 kΩ, consistent with an open circuit (Figure 1). This electrical instability accelerated battery consumption, and the device entered elective replacement indicator (ERI) mode within months of implantation—a highly unusual finding, as large registries report lead failure rates of <1% at one year. Notably, at previous follow-up visits, no signs of lead malfunction or abnormal impedance trends had been detected and, unlike implantable loop recorders, conventional dual-chamber pacemakers do not systematically provide continuous remote monitoring, and in this case no remote alerts were available. However, despite the abrupt impedance drop and premature ERI, no loss of capture, pauses, or syncope were documented before replacement.

**Figure 2:**
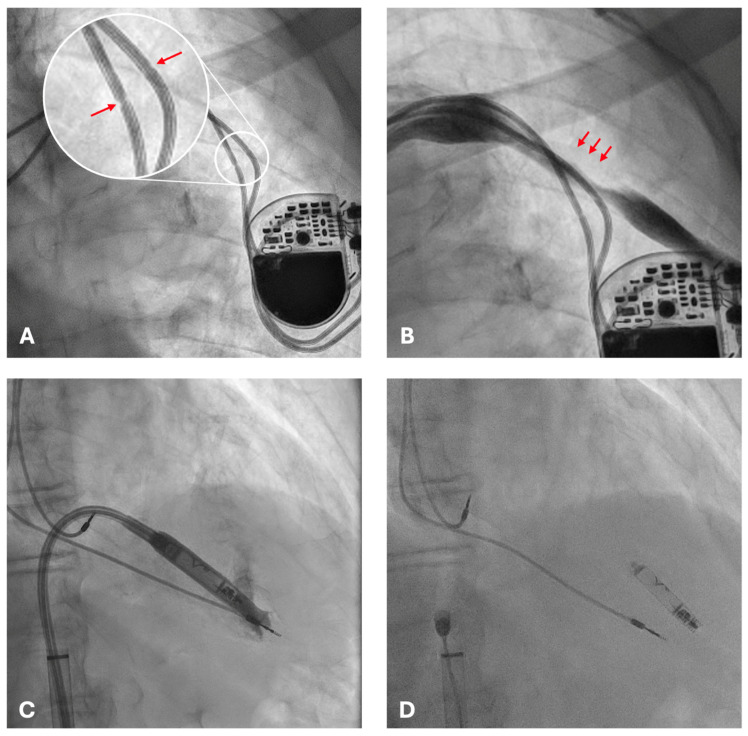
Fluoroscopic images during evaluation and intervention. (**A**) Dual-chamber pacemaker with atrial and ventricular leads in situ; arrows indicate focal narrowing of the leads near the clavicular region, consistent with mechanical compression injury from suture fixation without protective sleeves. (**B**) Distortion of the venous course with focal narrowing (arrows) along the left subclavian vein, consistent with complete left subclavian vein occlusion, which precluded new transvenous access. (**C**) Advancement of the leadless pacemaker delivery sheath into the right ventricle, with interference from pre-existing intracardiac leads. (**D**) Final stable deployment of the Aveir leadless pacemaker on the apico-septal region of the right ventricle, with satisfactory positioning and electrical performance despite the presence of abandoned leads.

Fluoroscopic examination demonstrated the pacemaker generator in situ with both atrial and ventricular leads displaying focal narrowings in the infraclavicular region (Figure 2A). These findings were consistent with chronic mechanical damage, most likely related to suture fixation without protective sleeves at the time of implantation.

Conventional transvenous reimplantation was not feasible because of complete left subclavian vein occlusion (Figure 2B) and arterial overlap. Extraction of the damaged leads was deemed high risk. Leadless pacemaker implantation represented the only feasible and safe alternative, given the presence of complete venous occlusion, the high hemorrhagic risk of contralateral reimplantation, and the prohibitive risk of extraction. A leadless pacemaker (Aveir, Abbott, Abbott Park, IL, USA) was therefore selected as the optimal solution.

The procedure was performed via ultrasound-guided right femoral venous access without interruption of anticoagulation therapy. The presence of pre-existing intracardiac leads posed technical challenges, obstructing advancement of the delivery system (Figure 2C) and requiring multiple repositioning attempts. After careful manipulation and sheath angulation adjustments, stable fixation of the leadless device was achieved on the apico-septal region of the right ventricle (Figure 2D).

Final pacing parameters were optimal (threshold 0.5 V at 0.4 ms, R-wave 10 mV, impedance 1070 w Ω). No procedural complications occurred, and no bleeding events were observed despite uninterrupted anticoagulation.

The patient underwent neurological rehabilitation with partial functional recovery. At discharge, his modified Rankin Scale (mRS) score was 2, indicating slight disability but preserved independence.

At two-months follow-up, the leadless device demonstrated stable function with excellent electrical performance, a ventricular pacing percentage of 41%, and the patient showed ongoing neurological recovery.

## 3. Discussion

Contemporary registries consistently show that leadless systems perform well even in higher-risk cohorts. In the 12-month Micra AV Post-Approval Registry (*n* = 801), implantation success was 99.4% and major complications were significantly lower than with transvenous dual-chamber systems (3.7% vs. 8.8%) [10]. Five-year data from the Micra post-approval registry also demonstrate durable safety with low revision and infection rates [11]. For dual-chamber leadless pacing, pivotal investigations of the AVEIR DR system reported very high implant success (98–99%) with robust atrioventricular synchrony and acceptable 1-year complication profiles [12,13].

What makes this case distinctive is the convergence of challenges: an abrupt lead-impedance drop–rise with premature ERI, device-detected weeks of AF coinciding with stroke, complete ipsilateral subclavian occlusion with abandoned leads obstructing delivery, and the strict requirement for uninterrupted anticoagulation after ischemic stroke. Such a combination has rarely been reported in prior series. Our report therefore expands current evidence by illustrating a real-world rescue strategy when multiple constraints intersect, a scenario not captured in registries or larger studies. It was this clustering of adverse factors—rather than any single element—that defined the therapeutic complexity and guided management. Notably, the most striking feature was the abrupt failure of a dual-chamber system within only one year of implantation, an exceptionally early event compared with expected device longevity. In large national registries, lead failure rates during the first year remain well below 1%, with most malfunctions occurring after several years [14,15]. This strongly suggests a preventable technical cause rather than intrinsic device defect.

The fluoroscopic finding of focal narrowings along the lead trajectory supports the hypothesis of suture-related compression injury. Such injury is avoidable with meticulous implant technique and routine use of protective sleeves [16,17]. Preventive strategies include systematic use of protective sleeves at all tie-down points, careful adjustment of suture tension to avoid focal compression, preference for axillary or cephalic venous access to minimize costoclavicular crush, intra-procedural fluoroscopic and impedance checks, and structured operator training with procedural checklists. Remote monitoring may also help to detect abrupt impedance shifts earlier.

Another central message is the value of systematic device interrogation. In this patient, pacemaker checks did not merely reveal technical malfunction but also uncovered an elevated arrhythmic burden and several weeks of persistent atrial fibrillation preceding the ischemic stroke. Interrogation thus provided crucial diagnostic information, suggesting a possible etiological link between arrhythmia and the neurological event [18]. However, although persistent AF preceded the ischemic event in our patient, the temporal association does not prove causality. Observational data have linked device-detected AF to increased stroke risk but with relatively low absolute event rates [19]. Randomized trials in subclinical AF show mixed results: apixaban reduced stroke versus aspirin at the expense of more major bleeding [20], whereas edoxaban did not reduce a composite ischemic endpoint and increased bleeding [21]. These findings support our cautious interpretation and individualized anticoagulation strategy rather than a definitive causal claim. This highlights how device data can guide stroke prevention strategies, especially in patients with elevated CHA_2_DS_2_-VASc scores, for whom guideline-directed anticoagulation is essential.

In our case, the therapeutic pathway was also shaped by anatomic and procedural challenges. Conventional transvenous reimplantation was not feasible because of venous occlusion and arterial overlap, while extraction was judged high risk [22]. Contralateral transvenous reimplantation was avoided because creation of a new pocket and venous puncture under uninterrupted apixaban therapy, mandated after the recent ischemic stroke, carried a substantial risk of pocket hematoma and subsequent infection. Leadless pacing provided a feasible alternative [23]. Despite technical difficulties from intracardiac obstacles, the Aveir device was successfully implanted, restoring ventricular pacing with stable parameters. Anticoagulation by itself is not an indication for leadless pacing. In this case, however, the combination of complete ipsilateral venous occlusion, the need to avoid lead extraction, and the requirement for uninterrupted oral anticoagulation after stroke made the femoral leadless approach the most appropriate strategy.

**Figure 3:**
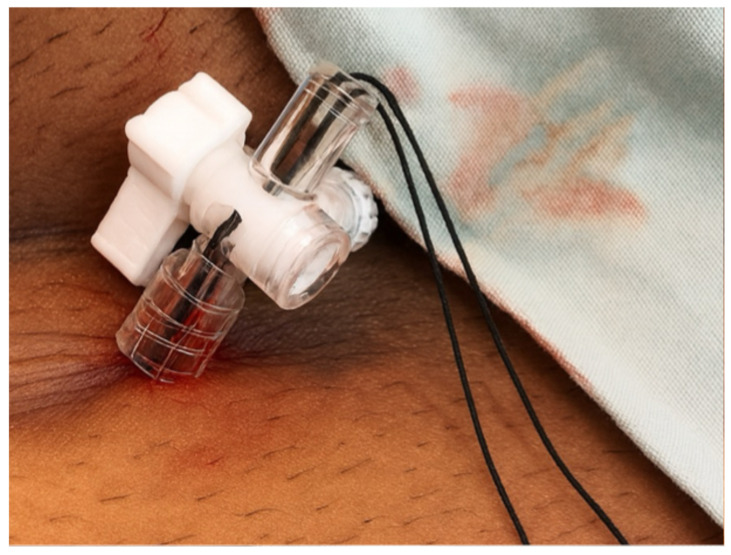
Femoral access closure technique using a figure-of-eight suture fixed inside a 3-way stopcock. The suture is passed subcutaneously at the puncture site and secured within the stopcock, which allows constant adjustable compression over 24 h, thereby minimizing hematoma risk while enabling uninterrupted oral anticoagulation.

Finally, the use of femoral venous access offered an important advantage in this anticoagulated patient [24]. Unlike subclavian or axillary approaches, the femoral route avoids creation of a subcutaneous pocket and reduces the risk of clinically significant bleeding or hematoma. Registry data confirm the safety of uninterrupted anticoagulation during leadless implantation, making this strategy particularly suitable for patients with recent ischemic stroke in whom strict maintenance of anticoagulation is mandatory [25,26]. At our center we perform femoral haemostasis by means of a figure-of-eight suture, closing the suture point inside a 3-way tap (Figure 3). This technique allows constant compression at the access level for 24 h, thereby minimizing the risk of hematoma formation. Combined with the absence of a subcutaneous pocket, this approach reduces bleeding complications and allows safe continuation of oral anticoagulation.

Follow-up is currently limited to two months, which precludes firm conclusions on long-term stability of the leadless system in the presence of abandoned leads. The patient is scheduled for device interrogations at 6 and 12 months, which will provide further insight into durability and safety. It should also be noted that this report reflects a single patient with short-term follow-up, which limits generalizability. However, while device interrogation strongly suggested a temporal association between atrial fibrillation and ischemic stroke, causality cannot be definitively established. Moreover, the absence of remote monitoring restricted continuous arrhythmia detection and may have delayed recognition of lead malfunction.

## 4. Conclusions

What makes this case particularly noteworthy is the simultaneous occurrence of multiple adverse factors, rarely reported together in the literature. Furthermore, it underscores that even minor technical oversights at pacemaker implantation may result in serious complications within an unusually short timeframe. Device interrogation proved invaluable, simultaneously identifying lead malfunction and clarifying arrhythmic burden, thereby informing both technical management and stroke prevention. Leadless pacing via femoral access emerged as a safe and effective rescue strategy in a patient requiring uninterrupted anticoagulation. As the population of anticoagulated and anatomically complex patients continues to grow, leadless systems are likely to play an increasingly central role in contemporary pacing practice.

## Data Availability

The original contributions presented in this study are included in the article. Further inquiries can be directed to the corresponding author.

## Abbreviations

The following abbreviations are used in this manuscript:

AF	Atrial Fibrillation
NIHSS	National Institutes of Health Stroke Scale
ERI	Elective Replacement Indicator
RRT	Recommended Replacement Time
mRS	Modified Rankin Scale

## References

[1] Glikson M., Nielsen J.C., Kronborg M.B., Michowitz Y., Auricchio A., Barbash I.M., Barrabés J.A., Boriani G., Braunschweig F., Brignole M. (2021). 2021 ESC Guidelines on cardiac pacing and cardiac resynchronization therapy. Eur. Heart J..

[2] Kusumoto F.M., Schoenfeld M.H., Barrett C., Edgerton J.R., Ellenbogen K.A., Gold M.R., Goldschlager N.F., Hamilton R.M., Joglar J.A., Kim R.J. (2019). 2018 ACC/AHA/HRS Guideline on the Evaluation and Management of Patients With Bradycardia and Cardiac Conduction Delay: A Report of the American College of Cardiology/American Heart Association Task Force on Clinical Practice Guidelines and the Heart Rhythm Society. J. Am. Coll. Cardiol..

[3] Kirkfeldt R.E., Johansen J.B., Nohr E.A., Jørgensen O.D., Nielsen J.C. (2014). Complications after cardiac implantable electronic device implantations: An analysis of a complete, nationwide cohort in Denmark. Eur. Heart J..

[4] Schnorr B., Kelsch B., Cremers B., Clever Y.P., Speck U., Scheller B. (2010). Contemporary issues in cardiac pacing. Minerva Cardioangiol..

[5] Witkowski M., Bissinger A., Grycewicz T., Lubinski A. (2017). Asymptomatic atrial fibrillation in patients with atrial fibrillation and implanted pacemaker. Int. J. Cardiol..

[6] Van Gelder I.C., Rienstra M., Bunting K.V., Casado-Arroyo R., Caso V., Crijns H.J.G.M., De Potter T.J.R., Dwight J., Guasti L., Hanke T. (2024). 2024 ESC Guidelines for the management of atrial fibrillation developed in collaboration with the European Association for Cardio-Thoracic Surgery (EACTS). Eur. Heart J..

[7] Chan N.Y., Kwong N.P., Cheong A.P. (2017). Venous access and long-term pacemaker lead failure: Comparing contrast-guided axillary vein puncture with subclavian puncture and cephalic cutdown. Europace.

[8] Xu F., Meng L., Lin H., Xu W., Guo H., Peng F. (2024). Systematic review of leadless pacemaker. Acta Cardiol..

[9] Mitacchione G., Schiavone M., Gasperetti A., Arabia G., Breitenstein A., Cerini M., Palmisano P., Montemerlo E., Ziacchi M., Gulletta S. (2023). Outcomes of leadless pacemaker implantation following transvenous lead extraction in high-volume referral centers: Real-world data from a large international registry. Heart Rhythm.

[10] Garweg C., Chinitz J.S., Marijon E., Haeberlin A., Winter S., Iacopino S., Curnis A., Breitenstein A., Hussin A., Mela T. (2024). A leadless ventricular pacemaker providing atrioventricular synchronous pacing in the real-world setting: 12-Month results from the Micra AV post-approval registry. Heart Rhythm.

[11] El-Chami M.F., Garweg C., Clementy N., Al-Samadi F., Iacopino S., Martinez-Sande J.L., Roberts P.R., Tondo C., Johansen J.B., Vinolas-Prat X. (2024). Leadless pacemakers at 5-year follow-up: The Micra transcatheter pacing system post-approval registry. Eur. Heart J..

[12] Doshi R.N., Ip J.E., Defaye P., Reddy V.Y., Exner D.V., Canby R., Shoda M., Bongiorni M.G., Hindricks G., Neuzil P. (2025). Dual-chamber leadless pacemaker implant procedural outcomes: Insights from the AVEIR DR i2i study. Heart Rhythm.

[13] Doshi R.N., Ip J.E., Defaye P., Exner D.V., Reddy V.Y., Hindricks G., Canby R., Shoda M., Bongiorni M.G., Neužil P. (2025). Chronic wireless communication between dual-chamber leadless pacemaker devices. Heart Rhythm.

[14] Sterns L.D. (2019). Pacemaker lead surveillance and failure: Is there a signal in the noise?. Heart Rhythm.

[15] Haeberlin A., Anwander M.T., Kueffer T., Tholl M., Baldinger S., Servatius H., Lam A., Franzeck F., Asatryan B., Zurbuchen A. (2021). Unexpected high failure rate of a specific MicroPort/LivaNova/Sorin pacing lead. Heart Rhythm.

[16] Bansal R., Kumar V., Talwar K.K. (2018). Traumatic Fracture of Pacemaker Lead by Suture Transfixation to Pectoral Muscle. J. Invasive Cardiol..

[17] Rezazadeh S., Wang S., Rizkallah J. (2019). Evaluation of common suturing techniques to secure implantable cardiac electronic device leads: Which strategy best reduces the lead dislodgement risk?. Can. J. Surg..

[18] Al-Gibbawi M., Ayinde H.O., Bhatia N.K., El-Chami M.F., Westerman S.B., Leon A.R., Shah A.D., Patel A.M., De Lurgio D.B., Tompkins C.M. (2021). Relationship between device-detected burden and duration of atrial fibrillation and risk of ischemic stroke. Heart Rhythm.

[19] Healey J.S., Connolly S.J., Gold M.R., Israel C.W., Van Gelder I.C., Capucci A., Lau C.P., Fain E., Yang S., Bailleul C. (2012). Subclinical atrial fibrillation and the risk of stroke. N. Engl. J. Med..

[20] Healey J.S., Lopes R.D., Granger C.B., Alings M., Rivard L., McIntyre W.F., Atar D., Birnie D.H., Boriani G., Camm A.J. (2024). Apixaban for Stroke Prevention in Subclinical Atrial Fibrillation. N. Engl. J. Med..

[21] Kirchhof P., Toennis T., Goette A., Camm A.J., Diener H.C., Becher N., Bertaglia E., Blomstrom Lundqvist C., Borlich M., Brandes A. (2023). Anticoagulation with Edoxaban in Patients with Atrial High-Rate Episodes. N. Engl. J. Med..

[22] Li X., Ze F., Wang L., Li D., Duan J., Guo F., Yuan C., Li Y., Guo J. (2014). Prevalence of venous occlusion in patients referred for lead extraction: Implications for tool selection. Europace.

[23] Darlington D., Brown P., Carvalho V., Bourne H., Mayer J., Jones N., Walker V., Siddiqui S., Patwala A., Kwok C.S. (2022). Efficacy and safety of leadless pacemaker: A systematic review, pooled analysis and meta-analysis. Indian Pacing Electrophysiol. J..

[24] Jelisejevas J., Breitenstein A., Hofer D., Winnik S., Steffel J., Saguner A.M. (2021). Left femoral venous access for leadless pacemaker implantation: Patient characteristics and outcomes. Europace.

[25] Oliveira S.F., Carvalho M.M., Adão L., Nunes J.P. (2021). Clinical outcomes of leadless pacemaker: A systematic review. Minerva Cardiol. Angiol..

[26] Kadado A.J., Chalhoub F. (2023). Periprocedural anticoagulation therapy in patients undergoing micra leadless pacemaker implantation. Int. J. Cardiol..

